# Quantum confinement of the Dirac surface states in topological-insulator nanowires

**DOI:** 10.1038/s41467-021-21230-3

**Published:** 2021-02-15

**Authors:** Felix Münning, Oliver Breunig, Henry F. Legg, Stefan Roitsch, Dingxun Fan, Matthias Rößler, Achim Rosch, Yoichi Ando

**Affiliations:** 1 Physics Institute II, University of Cologne, Zülpicher Str. 77, 50937 Köln, Germany; 2 Institute for Theoretical Physics, University of Cologne, Zülpicher Str. 77, 50937 Köln, Germany; 3 Department of Physics, University of Basel, Klingelbergstrasse 82, CH-4056 Basel, Switzerland; 4 Institute of Physical Chemistry, University of Cologne, Luxemburger Str. 116, 50939 Köln, Germany

**Keywords:** Nanowires, Electronic devices, Topological insulators

## Abstract

The non-trivial topology of three-dimensional topological insulators dictates the appearance of gapless Dirac surface states. Intriguingly, when made into a nanowire, quantum confinement leads to a peculiar gapped Dirac sub-band structure. This gap is useful for, e.g., future Majorana qubits based on TIs. Furthermore, these sub-bands can be manipulated by a magnetic flux and are an ideal platform for generating stable Majorana zero modes, playing a key role in topological quantum computing. However, direct evidence for the Dirac sub-bands in TI nanowires has not been reported so far. Here, using devices fabricated from thin bulk-insulating (Bi_1−*x*_Sb_*x*_)_2_Te_3_ nanowires we show that non-equidistant resistance peaks, observed upon gate-tuning the chemical potential across the Dirac point, are the unique signatures of the quantized sub-bands. These TI nanowires open the way to address the topological mesoscopic physics, and eventually the Majorana physics when proximitized by an *s*-wave superconductor.

## Abstract (web summary)

In topological insulator nanowires quantized Dirac sub-bands are expected, but direct evidence is still missing. Here, the authors report signatures of sub-bands in the gate-voltage dependence of the resistance by tuning the chemical potential in (Bi_1−x_Sb_x_)_2_Te_3_ nanowires through the Dirac point.

## Introduction

**Fig. 1:**
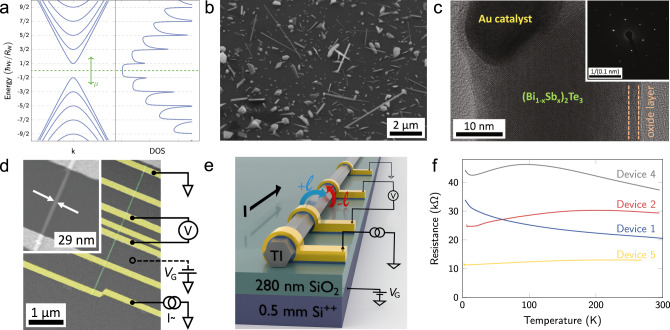
Topological-insulator nanowire and its device. **a** The sub-band structure of quantum-confined TI surface states described by Eq. (1) (left) and corresponding density of states (right). **b** SEM image of nanowires (and other nanostructures) grown on a substrate. **c** TEM image revealing the single-crystalline nanowire body, the remainder of the 20 nm Au-nanoparticle growth catalyst, and a 4-nm thick oxide layer at the nanowire surface. The inset shows the SAED pattern taken in the *c*-axis direction. TEM image and SAED pattern were taken from different nanowires and directions. **d** false-color SEM micrograph of device 5 with the schematics of electrical wiring. Pt/Au leads are colored yellow, the nanowire is shown in green. The inset shows a magnified view of the nanowire. **e** Schematic 3D image of the device construction. Pt/Au leads are illustrated in yellow. The blue and red arrow illustrate the angular momentum *ℓ*. **f ***R*(*T*) curves of devices 1, 2, 4, and 5.

In topological insulator (TI) nanowires^1–3^, the quantum confinement of the electron motion along the circumferential direction is described by the angular-momentum quantum number *ℓ*. In zero magnetic field, this quantization leads to the gap opening at the Dirac point, and the sub-bands become doubly-degenerate (see Fig. 1a). When a magnetic flux Φ threads along the wire, the energy spectrum is modified in a nontrivial way as described by the following formula (under the simplified assumption of a circular wire cross-section):

1 $${E}_{\ell }(k)=\pm \hslash {v}_{{\rm{F}}}\sqrt{{k}^{2}+{\left(\frac{\ell -\eta }{{R}_{{\rm{w}}}}\right)}^{2}},\quad \eta \equiv \Phi /{\Phi }_{0}.$$

Here, *v*_F_ is the Fermi velocity, *R*_w_ is the wire radius, and Φ_0_ = *hc*/*e* is the flux quantum; note that *ℓ* takes half-integer values $\pm \!\frac{1}{2},\pm \!\frac{3}{2},\ldots\!$ due to a Berry phase arising from the spin-momentum locking of the TI surface states^1^. Interestingly, a spin-non-degenerate gapless spectrum is restored when Φ is a half-integer multiple of Φ_0_; the spin-momentum locking in this gapless sub-band leads to the appearance of Majorana zero modes (MZMs) when the wire is proximitized by an *s*-wave superconductor^3,4^. The tunability of the spin-momentum locking with Φ makes the sub-bands described by Eq. (1) a particularly interesting platform for topological mesoscopic physics.

In experiments, to elucidate the peculiar quantization effects, the TI nanowire should be bulk-insulating and as narrow as possible, preferably less than ~ 100 nm. Past efforts for TI nanowires^5–16^ have only been able to indirectly probe the quantized Dirac sub-bands, although bulk-insulating TI nanowires have been occasionally reported^12,16–21^. In this work, we employed the vapor–liquid–solid (VLS) method using Au nanoparticles as catalysts^5^ and applied the concept of compensation, which has been useful for achieving bulk-insulation in bulk crystals^22,23^. Specifically, we tuned the Bi/Sb ratio of ${({{\rm{Bi}}}_{1-{\rm{x}}}{{\rm{Sb}}}_{{\rm{x}}})}_{2}{{\rm{Te}}}_{3}$ nanowires to a value that yields the most insulating properties. Fabrication of gate-tunable four-terminal devices allows us to bring the chemical potential across the Dirac point, upon which we discovered unusual oscillatory behavior in the resistance near the Dirac point in very thin wires. This feature turns out to be the signature of the quantized Dirac sub-bands in TI nanowires as our theoretical calculations show.

## Results

### Structural and chemical analysis

During the VLS growth, the catalysts form a constantly over-saturated liquid alloy with the absorbed source materials, which then precipitate and form a crystal underneath. Using nominally-20-nm-diameter Au nanoparticles as catalysts, we obtain nanowires with a constant diameter between 20 and 100 nm, with a length of up to several *μ*m (Fig. 1b). By using transmission electron microscopy (TEM) and energy-dispersive X-ray (EDX) analysis (Fig. 1c), we identify the Au catalyst at the tip of most of the analyzed nanowires. The wires are found to be surrounded by a ~4-nm-thick amorphous oxide shell. The selected-area diffraction patterns (SAED, Fig. 1c inset) indicate a high crystalline quality. We found hexagonal symmetry for a direction perpendicular the nanowire axis, which allows us to identify the growth direction to be $\langle 11\bar{2}0\rangle$-type. The compositional analysis using EDX along the wire shows a constant stoichiometry (Bi_0.68_Sb_0.32_)_2_Te_3_ in the nanowire core and no incorporation of Au was detected (See Supplementary Note 2).

### Temperature and gate-dependent device resistance

**Fig. 2:**
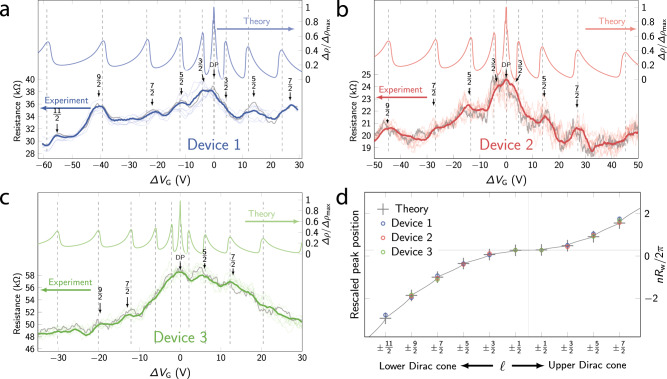
Signature of sub-band crossings. In panels **a**-**c**, lower curves show the *V*_G_ dependence of *R* observed in devices 1-3 at 2 K; Δ*V*_G_ = 0 corresponds to the Dirac point of the TI surface state, which was achieved with *V*_G_ of 30.5, 10, and 18.5 V in devices 1, 2, and 3, respectively. For each device multiple consecutive sweeps (9, 54, and 40 for devices 1, 2, and 3, respectively) were performed. For clarity, only 9 of these sweeps are shown for each device as thin lines, together with a single exemplary curve highlighted in bold gray. From the average (colored bold lines) large oscillations of type I are identified (see main text) which arise from the sub-band structure and the corresponding maxima are labeled by arrows. Oscillations of type II are smaller and differ between individual sweeps. Upper curves in **a**–**c** are the theoretically calculated resistivity with a small density of impurities, assuming that electron density *n* is proportional to *V*_G_; we used *R*_w_ of 20, 16, and 20 nm, and *C*_G_ of 2.0, 2.2, and 3.6 pF/m for devices 1, 2, and 3, respectively. Pronounced maxima arise at sub-band crossings (dashed lines). **d** Rescaled position Δ*V*_G_/*V*_0_ (see main text) of the resistivity maxima of the three devices (open circles) as a function of the quantum number *ℓ* compared to the theoretically calculated *n* in units of 2*π*/*R*_w_ (solid line). Crosses indicate the value of *n* at sub-band minima.

In the following, we report five representative devices 1–5. The scanning electron microscope (SEM) picture of device 5 is shown in Fig. 1d, with its schematic depicted in Fig. 1e. The single-crystalline nanowires are most likely of hexagonal shape (see Supplementary Note 1). The resistance *R* vs. temperature *T* curves shown in Fig. 1f present both insulating and metallic behavior; nevertheless, all these samples were bulk-insulating, which can be seen in their gate-voltage *V*_G_ dependences of *R* (Fig. 2a–c and Supplementary Fig. 4) showing a clear maximum, indicating that the Dirac point is crossed. The difference in the *R*(*T*) dependence is most likely explained by a slightly different electron density *n* of the samples in the absence of gating (*n* ≈ 0.38, 0.14, 0.42, − 0.2, 0.5 nm^−1^ relative to the Dirac point, according to our analysis described later).

In the *R*(*V*_G_) traces, we found a hierarchy of fluctuation features. We observe semi-oscillatory features in the *V*_G_ dependence with the amplitude *A*_I_ ≈ 3, 2 and 1 kΩ for devices 1, 2, and 3, respectively. We will show these features (type I) to be the signature of sub-band crossings. They are not universal conductance fluctuations (UCF) whose main fingerprint would be a strong change in magnetic field and the lack of any clear periodicity and a random amplitude. In contrast, the present features of type I occur in a regular fashion, i.e., at regularly spaced gate voltages and with a largely uniform amplitude. Further, they are robust in small applied magnetic fields (for a detailed discussion on UCF see Supplementary Note 3). The other type of fluctuations have the amplitude *A*_II_ ≈ 0.5 kΩ (type II) and were changing with time (Supplementary Fig. 3). We speculate that they arise from time-dependent conductance fluctuations due to charge traps or mobile scattering centers, similar to those observed in metallic nanowires of similar mesoscopic size^24^, but they may also be affected by the presence of electron-hole puddles^25–27^. Averaging over several gate-voltage sweeps suppresses type II fluctuations while type I fluctuations remain unaffected, see Supplementary Note 4.

### Model of gate-voltage dependent surface conduction

**Fig. 3:**
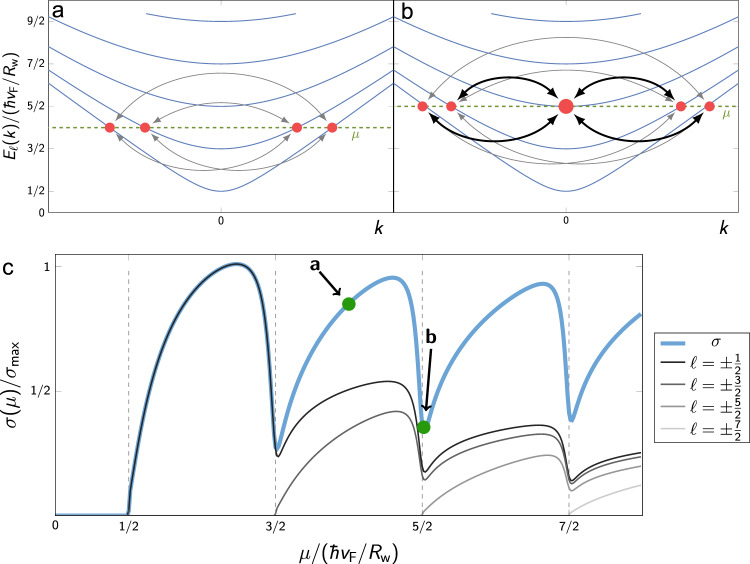
Scattering processes and conductivity. **a, b** Scattering processes (arrows) between conduction channels for two positions of *μ* (dashed line). When *μ* is at the bottom of a sub-band, **b** all other sub-bands scatter at large rates (bold arrows) with the new sub-band due to its diverging density of states (illustrated by a large red dot), leading to a pronounced minimum in the conductivity. **c** Theoretically-calculated conductivity as function of *μ* (parameters as in Fig. 2). Thin black lines display the contribution of each sub-band labeled by $\ell =\pm \!\frac{1}{2},\pm \!\frac{3}{2},\pm \!\frac{5}{2},\pm \!\frac{7}{2}$, which add up to give the total conductivity (thick blue line). The conductivity of all channels shows pronounced minima at *μ* = *ℓ*ℏ*v*_F_/*R*_w_, when the chemical potential touches the bottom of a new sub-band.

We now discuss the main observation of this work, that is, the reproducible semi-oscillatory feature in the *R*(*V*_G_) curves. Due to the 1D nature of the energy bands in the nanowire, the density of states (DOS) diverges as $1/\sqrt{E}$ at each of the sub-band’s edges as shown in Fig. 1a. This causes a sub-band crossing to have two contrasting effects on *R*: (i) The opening of a new conductance channel can decrease the resistivity as more charge can be transported. It can, however, also (ii) increase the resistivity by opening a new channel where electrons from other bands can scatter into. Thus, we have performed a straightforward theoretical calculation using an idealized model based on the surface state of a circular TI nanowire. (Small anisotropy effects arising both from the hexagonal shape of the wire and the anisotropic electrostatic environment are discussed in the Supplementary Notes 13–16). The effects of local impurities are taken into account using the T-matrix formalism and we find that the experimental data is best described by weak impurities (see Supplementary Note 11). In Fig. 3, we schematically show how different sub-bands contribute to the conductivity: When a new channel is added (Fig. 3b), all other channels scatter efficiently into the new channel and, as a result, the conductivity contribution of each channel drops. This is by far the dominant effect and leads to pronounced peaks in *R* even when several channels are present. The diverging density of states of the newly added channel (Fig. 1a) is the main reason why this effect is so large, but it is further enhanced by a matrix-element effect originating in the topological protection of the surface states (Supplementary Note 11).

Hence, our calculations show that the resistance is expected to show a peak, each time a sub-band is crossed. This leads to equidistant peaks in Fig. 3, at *μ* = *ℓℏv*_F_/*R*_*w*_, when the conductivity is plotted as function of the chemical potential *μ*. In the experiment, however, the gate voltage *V*_G_, rather than *μ*, is varied and we observe a super-linear dependence of the spacings of the main peaks (neglecting features of type II). This originates from the fact that the effective capacitance of the nanowire devices (which dictates the *V*_G_ dependence of the accumulated charge) must be computed from their quantum capacitance *C*_Q_ and geometric (or galvanic) capacitances *C*_G_ in series where *C*_Q_ is proportional to the DOS^16^. In our experiment, *C*_G_ strongly dominates and the gate voltage directly controls the electron density *n* (*n* ≈ *C*_*G*_Δ*V*_G_/*e*, with Δ*V*_G_ measured from the Dirac point), rather than *μ*. This relation is used for the theory plots in Fig. 2. It also determines the peak positions indicated by dashed lines. We label the position of the peaks identified in the experimental data by the angular momentum quantum number *ℓ* of the added channel. The influence of the flat bottom gate geometry on the charge homogeneity around the wire is negligible, since it does not affect the position of peaks due to Klein-tunneling physics^16^ (see Supplementary Note 13). When the chemical potential reaches the bottom of the first electron or the top of the first hole band ($\ell =\!\pm\! \frac{1}{2}$), the charge density is approximately zero in both cases and therefore there is only a single peak in the center for $\ell =\!\pm \!\frac{1}{2}$. For large *ℓ*, the peak position scales with *ℓ*^2^, which is peculiar to the sub-bands of Dirac origin, where the charge density grows as *μ*^2^ due to the 2D nature of the TI surface.

### Sub-band crossings observed in experiment

It is striking that in Figs. 2a–c the theory can reproduce the essential features of our experiment, in particular the locations of the peaks in the averaged *R*(*V*_G_) curves. While for devices 1 and 2 every peak can be indexed, type II features arising from disorder are more pronounced in device 3, such that some of the sub-band crossing features are not discernible despite averaging. To visualize the agreement between theory and experiment, we plot in Fig. 2d the rescaled gate voltage values of the peaks, Δ*V*_G_/*V*_0_, vs the sub-band index *ℓ*, and compare it to the theoretically calculated electron density *n* at the peak position (in units of 2*π*/*R*_w_). In these units the rescaling factor is given by ${V}_{0}=\frac{2\pi e}{{R}_{{\rm{w}}}C}$, where *C* is the capacitance per length of the wire. The super-linear behavior in the *V*_G_-dependent sub-band crossings and the excellent agreement of theory and experiment is a direct signature of the quantum-confined Dirac surface states, which is observed here for the first time.

It is prudent to mention that the quantum-confined sub-band structure of TI nanowires have been indirectly inferred^5–16^ from the Aharonov–Bohm (AB)-like oscillations of *R* as a function of the axial magnetic flux Φ, which is due^1^ to a periodic change in the number of occupied sub-bands at a given *μ*. In particular, the observation by Cho et al.^12^ that *R* at Φ = 0 takes a maximum when *μ* is near the Dirac point and changes to a minimum at some other *μ* was consistent with the gapped Dirac cone; however, the *V*_G_ dependence was not very systematic nor convincing in ref. ^12^. A relatively systematic *V*_G_ dependence of *R* was recently reported for HgTe nanowires and was carefully analyzed^16^; unfortunately, the Dirac point of HgTe is buried in the bulk valence band, hindering the characteristic super-linear behavior in the Δ*V*_G_ vs *ℓ* relation from observation.

## Discussion

The realization of very thin, bulk-insulating TI nanowires and the observation of the quantum-confined Dirac sub-band structure reported here is crucial for exploring the mesosocpic physics associated with the topological surface states, not to mention their potential for future studies of MZMs. For example, the dependence of the spin degeneracy on the magnetic flux along the nanowires will give us a new tuning knob for mesoscopic transport phenomena, in which the spin-momentum locking can be varied. Also, it is an interesting insight that the charge inhomogeneity induced by gating on TI nanowires will not affect the energy locations of the sub-band crossings due to Klein-tunneling physics. Therefore, the new-generation TI nanowires realized here will open vast opportunities for future studies of topological mesoscopic physics including MZMs.

## Methods

### Nanowire synthesis

The ${({{\rm{Bi}}}_{1-{\rm{x}}}{{\rm{Sb}}}_{{\rm{x}}})}_{2}{{\rm{Te}}}_{3}$ nanowires were synthesized by the VLS method using powders of Bi_2_Te_3_ and Sb_2_Te_3_ as starting materials in a two-zone 50-mm tube-furnace under a constant Ar flow. The Si/SiO_2_ substrates were first decorated with suspended 20-nm Au-nanoparticles with the help of Poly-L-Lysine solution and then placed between the two zones (set to temperatures *T*_1_ and *T*_2_) of the furnace. The temperature was first ramped to *T*_1_ = 500–510 ^∘^C and *T*_2_ = 280 ^∘^C within 60 min, kept at these values for 60 min, and finally reduced back to ambient temperature in roughly 4 h, while keeping a constant Ar flow of 600 SCCM.

### Device fabrication

Our gate-tunable four-terminal devices were fabricated on degenerately-doped Si wafers covered by 280-nm thermally-grown SiO_2_ which acts as a gate dielectric. Gold contact pads and a coordinate system were pre-defined by optical lithography. The as-grown nanowires were transferred by gently bringing together the surfaces of the pre-patterned wafer and the growth substrate, and nanowires suitable for device fabrication were identified by optical microscopy. Per device, five to seven contacts with varying distances were defined by electron beam lithography, which was performed by exposing a PMMA A4 resist layer using a Raith PIONEER Two system. The contact area was cleaned using gentle oxygen plasma treatment and a dip in dilute hydrochloric acid shortly before metallization. Subsequently, 5-nm-thick Pt was sputter-deposited as a wetting layer and an additional 45-nm-thick Au layer was deposited by thermal evaporation (devices 4 & 5) or by sputtering (devices 1, 2, and 3), resulting in the structure schematically shown in Fig. 1e. The contact resistance was well below 1 kΩ for all of the devices. Following the transport measurements, SEM was used to determine the device geometry and the nanowire diameter. The distance between the centers of the voltage contacts were 0.5, 0.8, 1.0, 1.2, and 0.7 *μ*m, and the diameter of the nanowires were 41, 32, 41, 43, and 29 nm for devices 1–5, respectively.

### TEM analysis

TEM micrographs, as well as TEM diffraction patterns, were recorded by using a JEM 2200-FS (JEOL) microscope operated at an acceleration voltage of 200 kV. A carbon film supported by a standard copper grid was used as sample carrier for TEM characterization. Elemental chemical analysis of the samples was done by Energy-Dispersive X-ray Analysis (EDX) performed with a JEOL Dry SD100GV detector.

### Measurements

Transport measurements were performed in a liquid-helium cryostat in the temperature range of 2–300 K. The wafers were glued onto copper sample holders and manually bonded with 50-*μ*m gold wires using vacuum-cured silver paste. For fast measurements, we used a quantum transport measurement system (SPECS Nanonis Tramea) in the low-frequency lock-in mode with the ac current of 100 nA at the frequency *f* ≈ 17 Hz, while the device is configured in a conventional four-(device 3, 4, & 5) or three-(device 1 & 2) terminal geometry. Gate-voltage sweeps were performed at various rates from 0.0125 V/s to 0.25 V/s while monitoring the sample temperature with a dedicated thermometer using a low-power AC resistance bridge (Lakeshore Model 370).

### Theoretical calculations

We consider the surface states of a quantum wire described by the 2D Dirac equation where antiperiodic boundary conditions in the transverse direction arise from curvature-induced Berry phase effect^1^. Disorder is modeled by a small density of randomly located local scattering potentials, which is treated within a (non-self-consistent) T-matrix approximation, which can be calculated in a fully analytic way, see Supplementary Note 9. Within our approximation, qualitative features are independent of the density of impurities; however, they do depend on the amplitude of the scattering potential. The Kubo formula was used to calculate the conductivity. Vertex corrections were ignored as a previous study showed that they have only a small, purely quantitative effect^28^. Plots of resistivities were obtained from *ρ* = 1/(*σ*_0_ + *σ*(*μ*)), where *σ*_0_ is mainly used to avoid the divergence of *ρ* when *σ* = 0. It describes the presence of conductance contributions (e.g., from impurity bands on the surface or in the bulk) not taken into account in our approximation. Note that, although the experimental data are shown in resistance *R*, the theory calculates the resistivity *ρ*, because the transport is assumed to be in the diffusive regime. The dependence of *μ* on Δ*V*_G_ is computed from *n*(*μ*) = *C*_G_Δ*V*_G_/*e*, where *n*(*μ*) is the electron density along the wire and *C*_G_ is treated as a fitting parameter. Full details of our calculations including a discussion of effects arising from deviations of the circular shape of the wire are given in Supplementary Notes 13–16.

## Supplementary information

Supplementary Information

## Data Availability

The experimental data that support the findings of this study are available in figshare with the identifier doi:10.6084/m9.figshare.13524050 (ref. ^29^).
