# Supplementary material for: Quantum confinement of the Dirac surface states in topological-insulator nanowires
Source: Nat Commun. 2021 Feb 15;12:1038. doi: 10.1038/s41467-021-21230-3 (PMC7884718; doi:10.1038/s41467-021-21230-3)
Supplement: Supplementary file 1 — Supplementary Information [file 41467_2021_21230_MOESM1_ESM.pdf]

Supplementary Information for "Quantum confinement of the Dirac surface states in topological-insulator nanowires"

Felix Münning,[1] Oliver Breunig,[1] Henry F. Legg,[2,3] Stefan Roitsch,[4]
Dingxun Fan,[1] Matthias Rößler,[1] Achim Rosch,[2] and Yoichi Ando[1]

[1] *Physics Institute II, University of Cologne,*
Zülpicher Str. 77, 50937 Köln, Germany

[2] *Institute for Theoretical Physics, University of Cologne,*
Zülpicher Str. 77, 50937 Köln, Germany

[3] *Present address: Department of Physics, University of Basel,*
Klingelbergstrasse 82, CH-4056 Basel, Switzerland

[4] *Institute of Physical Chemistry, University of Cologne,*
Luxemburger Str. 116, 50939 Köln, Germany

Supplementary Note 1. Growth direction and shape of nanowires

TEM investigations reveal that the growth direction of the nanowires is parallel to the $\langle 11\bar{2}0\rangle$ direction, i.e. perpendicular to the c axis of the crystal structure. Since in our TEM characterization the nanowires lie on the supportive carbon carrier, the $\langle 11\bar{2}0\rangle$ direction is always oriented perpendicular to the optical axis of the microscope. However, the rotation of the nanowires along the growth direction is arbitrary with respect to the support. As a consequence, different orientations perpendicular to the growth direction can be observed by TEM. The c-axis, revealing the distinguished hexagonal symmetry, can only be observed occasionally. This fact also suggests that our nanowires are closer to a cylindrical shape than a ribbon shape. In this regard, it has been theoretically shown that the energetically most favorable cross-sectional shape of Bi_2Se_3 and Bi_2Te_3 nanowires grown along the $\langle 11\bar{2}0\rangle$ direction is hexagonal due to the alignment of atoms in neighbouring quintuple-layers [1]. In fact, it was experimentally found that a narrow Bi_2Se_3 nanowire (diameter of $\sim$80 nm) grown along the $\langle 11\bar{2}0\rangle$ direction has a hexagonal cross-section [2]. Therefore, it is most reasonable to assume that the narrow $(Bi_{1-x}Sb_x)_2Te_3$ nanowires grown in this work also have a hexagonal cross-section, rather than a rectangular cross-section often assumed for wide nanowires [3].

Supplementary Note 2. Homogeneity and purity of nanowires

The chemical composition of the $(Bi_{1-x}Sb_x)_2Te_3$ nanowire body is homogeneous along the nanowire axis, such that the atomic fraction of each element measured by EDX on six different locations varies only within a few percent (Fig. S1). An incorporation of Au atoms can be excluded within the accuracy of the measurement.

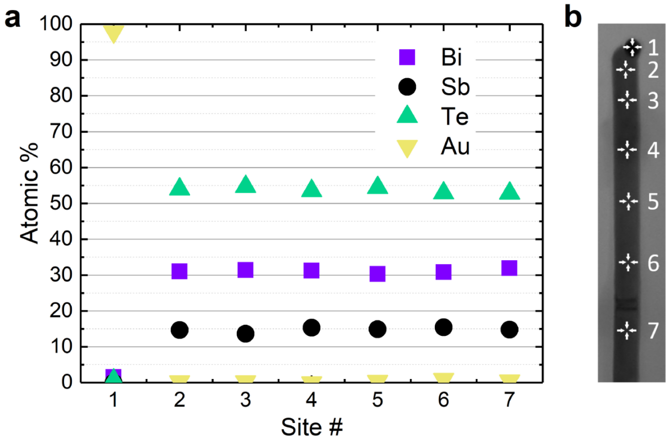

Supplementary Fig. 1: **Series of EDX measurements along the nanowire axis.** **a**, Atomic fraction of the elements Bi, Sb, Te, and Au at each location as shown in **b**.

Supplementary Note 3. Universal conductance fluctuations

In mesoscopic systems universal conductance fluctuations (UCF) arise from a specific distribution of scattering sites. Our devices fall into this regime, which raises the question whether type I peaks could also be explained by UCF. However, type I peaks are much too regular to be caused by conductance fluctuations. UCF occur without any clear periodicity as a function of gate voltage or magnetic field. In contrast, the features of type I that we identify with the sub-band crossings occur in a regular fashion, i.e. at regularly spaced gate voltages, as shown by Fig. 2d. Secondly, the amplitude of type I features strongly differs from what would be expected for typical UCF. For UCF, the amplitude of individual peaks is random. In contrast, our type I oscillations occur with a largely uniform amplitude. Both of these facts speak against UCF as the origin of type I peaks. Furthermore, for every type I peak at gate voltage ΔV_G we observe a symmetry-related peak close to $-\Delta V_G$ (see Fig. 2d). This is expected from the presence of an approximate particle-hole symmetry of the Dirac cone of $(Bi_{1-x}Sb_x)_2Te_3$ [4] and is not consistent with UCFs for which no such symmetry is expected.

Another hallmark of UCFs is that they are strongly affected by small magnetic fields. If features of type I in our gating curves were UCF, they should rapidly change when applying a small magnetic field. Using device 6 (fabricated in the same way to devices 1-5) multiple gate sweeps were performed at constant magnetic fields of 0.1 and 0.5 T. Fig. 2 shows the average of these sweeps (see Supplementary Note 4 below). While there is finite differences in the background (which partly consists of residual type II fluctuations that may well be field-dependent) the main features that we identify with the sub-band crossings remain robust upon changing the magnetic field. Thus, we can clearly rule out UCFs as the origin of type I peaks, both due to the regularity of their amplitude and periodicity, as well as their independence on small magnetic fields.

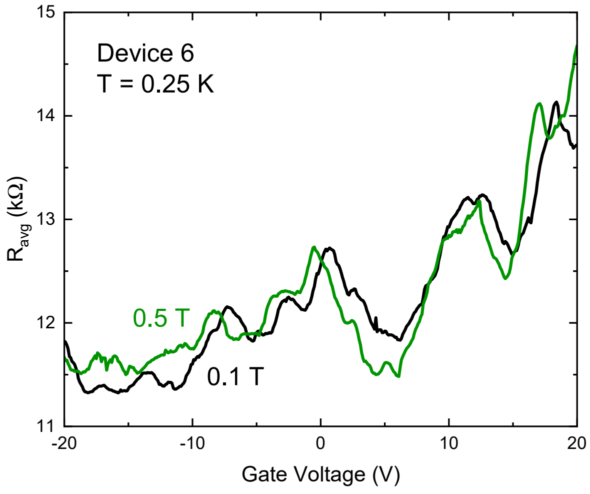

Supplementary Fig. 2: **Gate voltage dependence of the resistance of device 6.** The curves were obtained by averaging over multiple consecutive sweeps at magnetic fields of 0.1 and 0.5 T.

Supplementary Note 4. Averaging of multiple gate sweeps

As discussed in the main text, two distinct types of oscillations are seen in the $R(V_G)$ curves: Large oscillations coming from sub-band crossings (type I, ~ 3 kΩ) and smaller, time-dependent oscillations (type II, ~ 0.5 kΩ). The type-I oscillations are reproducible and most of the peaks are well pronounced. On the other hand, as shown examplarily for device 5 in Fig. 3, the resistance of the devices changes with a timescale of a few minutes. The magnitude of these jumps is of the order of the type II features in the $R(V_G)$ curves. We speculate that each jump of the resistance is associated with a reconfiguration of the scattering site distribution and thus changes the background on which we expect to observe the intrinsic gate voltage or magnetic field dependence of the surface state transport. Since the gating-curves were measured in about 80 minutes, this process also takes place during our gate sweeps. The fact that type II features consequently differ between each individual sweep opens a way to suppress their conductivity contribution by performing a time average or, more practically, an average of multiple sweeps. Comparing the individual gate sweeps with the average (see Fig. 2a-c of the main text) clearly shows that large features (type I) are preserved upon averaging while minor peaks (type II) in the individual curves are suppressed. As such, averaging of multiple sweeps is a useful tool to deterministically extract the dominant conductivity features due to the Dirac sub-band structure.

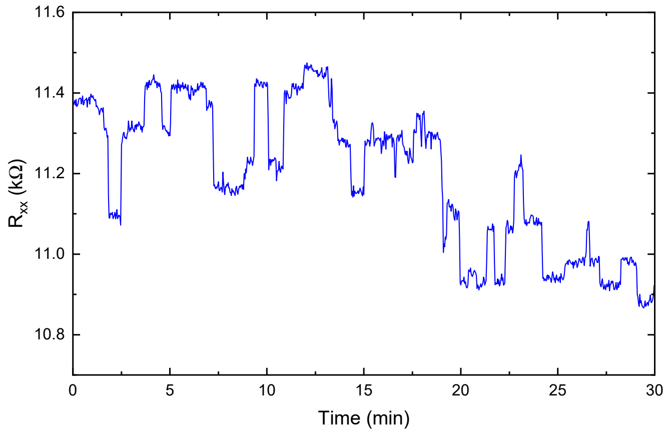

Supplementary Fig. 3: **Time-dependent resistance fluctuations.** Type II fluctuations measured as a function of time on device 5.

Supplementary Note 5. Identification of the sub-band peaks without averaging

Even without performing gate voltage sweep averages, type I features are also found in each individual sweep as discussed for two additionally fabricated devices in the following. For these devices we did not perform averaging at the time of the data acquisition. Yet, they also show full gate-tunability through the Dirac point and their individual gating curves exhibit features similar to those of devices 1–3 discussed in the main text (Supplementary Fig. 4). By comparison

to the theory, major peaks can be indexed for these devices as well, even without performing a time-consuming averaging over a large number of gate sweeps. However, the type-II oscillations can make the identification of the type-I peaks complicated, and in such rare cases it is useful to adopt two criteria for the identification of the type-I peaks: amplitude and regularity. Namely, we identify peaks that stand out in terms of the amplitude and appear semi-regularly to be type-I. For example, the identification of type-I peaks in device 5 (Supplementary Fig. 4b) followed these criteria. Nevertheless, we emphasize that in most cases (such as device 1 shown in Fig. 2a) the identification of type-I peaks is evident. The intrinsic type-I peaks identified this way remain robust after averaging multiple gate sweeps as mentioned above and turn out to agree well with the theoretical calculations as shown in the main text.

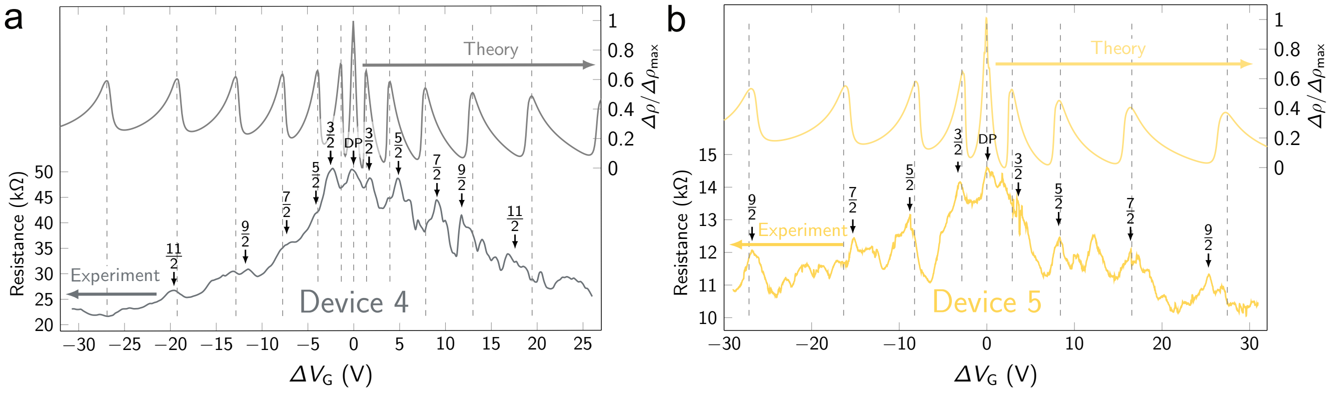

Supplementary Fig. 4: **Gate-voltage dependence of the resistance of the additional devices 4 and 5.** $\Delta V_{\rm G} = 0$ corresponds to the Dirac point of the TI surface state, which was achieved with $V_{\rm G}$ of -6 in device 4 and 19 V in device 5. We used $R_{\rm w}$ of 22 and 15 nm and $C_{\rm G}$ of 5.8 and 4.1 pF/m for devices 4 and 5, respectively. In comparison to theory, sub-band crossing peaks can be identified even though type II features are also present in these non-averaged, single gating curves.

Supplementary Note 6. Geometric and quantum capacitances

In general, the gate voltage required to provide the nanowire with additional charge Q can be expressed as

$$V_{\rm G} = \frac{Q}{C_{\rm G}} + \frac{Q}{C_{\rm Q}}, \tag{1}$$

where $C_{\rm G}$ is the geometric capacitance of the device and $C_{\rm Q}$ is the quantum capacitance, or, equivalently, as $eV_{\rm G} = \frac{e^2}{C_{\rm G}} n(\mu) - \mu$, where $e < 0$ is the electron charge, μ the chemical potential, and $n(\mu)$ the electron density. In our experiments, a change of the gate voltage by 30 V leads to changes of the chemical potential by less then 100 meV. Therefore, the effect of the geometric capacitance dominates by more than two orders of magnitude and we can safely approximate $V_{\rm G} \approx \frac{1}{C_{\rm G}} en(\mu)$.
The geometric capacitance $C_{\rm G}$ was found to be $2 - 3.6$ pF/m in our experiments. Note that $C_{\rm G}$ depends on materials properties and the device geometry, and one needs to numerically calculate it for each situation [5]. For the present gate geometry, as presented in the section Supplementary

Note 13, a value of $C_{\rm G} \approx 15$ pF/m would be expected. However, our nanowires are not metallic as is typically assumed in simulations, and hence the result of such numerical simulations should be taken as an upper bound for $C_{\rm G}$. In addition, the effective capacitance is further affected by parasitic capacitances due to the leads, as well as by the finite length of the nanowire. For these reasons we treat the value of the capacitance as a phenomenological parameter.

Supplementary Note 7. Hamiltonian of a disordered TI nanowire

The Hamiltonian for the surface of a cylindrical TI nanowire of radius R_w depends only on momentum around the wire, $\hat{p}_\phi = \hat{L}_\phi/R_w = -iv_F\partial_\phi/R_{\rm w}$, and momentum along the wire, $\hat{p}_x = -i\hbar\partial_x$, and is given by [6]

$$H_{\rm surf} = i\hbar v_F \left(\frac{\sigma_x}{R_{\rm w}} \partial_\phi - (\sin(\phi)\sigma_z - \cos(\phi)\sigma_y)\partial_x \right). \tag{2}$$

This can be simplified by applying a spinor-rotation by ϕ about the x-axis, *i.e.*, the unitary transformation $U_x(\phi) = \exp(-i\phi\sigma_x/2)$. The result is the Hamiltonian

$$H_0 = i\hbar v_F \left(\frac{\sigma_x}{R_{\rm w}} \partial_\phi - \sigma_y \partial_x \right). \tag{3}$$

In the presence of a flux Φ threaded along the wire, ∂_ϕ is replace by $\partial_\phi - i\eta$ with $\eta = \Phi/\Phi_0$. Importantly, since spinor rotations are 4π periodic, the eigenfunctions of this Hamiltonian must obey anti-periodic boundary conditions, resulting from $U_x(\phi) = -U_x(\phi + 2\pi)$. As such the wave function can be written in the general form $\psi_\ell(k) = e^{i(kx+\ell\phi)}\boldsymbol{\xi}$, where the anti-periodic boundary conditions require the total angular momentum to satisfy $\ell = \pm 1/2, \pm 3/2, \ldots$ and $\boldsymbol{\xi}$ is a spinor encoding the spin-structure of the surface state within this rotated system.

Due to the finite geometry along the circumference of the wire the eigenenergies of the Hamiltonian are quantised

$$E_{\ell,\pm}(k) = \pm\hbar v_F \sqrt{k^2 + \left(\frac{\ell - \eta}{R_{\rm w}} \right)^2}. \tag{4}$$

The band edge of each band is for $\eta = 0$ given by $\varepsilon_{\ell,\pm} = \pm\hbar v_F \ell/R_{\rm w}$. We define $\varepsilon_\ell = \varepsilon_{\ell,+}$.

Disorder is modelled by impurity potentials located at random positions $\boldsymbol{r}_i$, $V_i(\boldsymbol{r}) = u_0 \mathbb{1}\delta(\boldsymbol{r} - \boldsymbol{r}_i)$, where $\boldsymbol{r}$ and $\boldsymbol{r}_i$ are 2d coordinates on the surface of the wire.

Supplementary Note 8. Density of states and charge density

The free retarded Green's function – which is a matrix – has the form

$$[\boldsymbol{G}^0]_{\alpha\beta}(\mu, k, \ell) = (\mu\mathbb{1} - H_0(k,\ell) + i\delta\mathbb{1})^{-1}_{\alpha\beta}, \tag{5}$$

where we take the matrix inverse. Here δ acts as some overall background scattering rate due degrees of freedom (e.g. impurity bands) not described by our Hamiltonian. It has the effect of broadening the divergence of the density of states at the bottom of each sub-band.

The local Green's function of each ℓ resolved sub-band is given by

$$\mathbf{\Delta}(\mu,\ell) = \frac{1}{2\pi R_{\mathrm{w}} v_F}\int \frac{dk}{2\pi\hbar}\boldsymbol{G}^0(\mu,k,\ell) = -\frac{(\mu+i\delta)\mathbb{1} - \varepsilon_\ell \sigma_x}{4\pi v_F R_{\mathrm{w}}\hbar\sqrt{\varepsilon_\ell^2 - (i\delta+\mu)^2}}. \tag{6}$$

From the Green's function we can obtain the local density of states (at a fixed position at the surface of the wire)

$$\rho(\mu) = -\frac{1}{\pi}\sum_{\ell=\pm\frac{1}{2},\pm\frac{3}{2},\dots} \mathrm{Im}\left\{\mathrm{Tr}\ \mathbf{\Delta}(\mu,\ell)\right\} = \frac{1}{2\pi^2 R_{\mathrm{w}} v_F \hbar}\sum_{\ell=\pm\frac{1}{2},\pm\frac{3}{2},\dots}\mathrm{Im}\{\frac{\mu+i\delta}{\sqrt{\varepsilon_\ell^2-(i\delta+\mu)^2}}\}, \tag{7}$$

which, as should be expected, tracks the 2d Dirac density of states $\rho^{\mathrm{2d}}(\mu) = |\mu|/2\pi v_F^2\hbar^2$.

From the density of states we can also obtain the charge density along the wire. It is given by

$$n(\mu) = 2\pi R_{\mathrm{w}}\int_0^\mu d\mu'\rho(\mu') = -\frac{1}{\pi v_F \hbar}\mathrm{Im}\left\{\sum_{\ell=\pm\frac{1}{2},\pm\frac{3}{2},\dots}\sqrt{\varepsilon_\ell^2-(\mu+i\delta)^2}\right\}. \tag{8}$$

Here $n(\mu)$ is zero at the Dirac point and counts the total charge density per length of wire. For $\mu > 0$ this means counting the contribution of all bands above the Dirac point up to the chemical μ. Correspondingly, for $\mu < 0$ counting the charge of all holes contributed by bands below the Dirac point, in other words $n(\mu)$ is negative for $\mu < 0$. It is shown as a grey line in Fig. 2d of the main text.

Supplementary Note 9. T-matrix approximation

The full disorder averaged Green's function is

$$[\boldsymbol{G}]_{\alpha\beta}(\mu,k,\ell) = (\mu\mathbb{1} - H_0(k,\ell) + \mathbf{\Sigma}(\mu))^{-1}_{\alpha\beta}., \tag{9}$$

where $\mathbf{\Sigma}(\mu)$ is the full self-energy from all scattering processes, including disorder and the other scattering processes modelled by the factor $i\delta$ (see Supplementary Note 8).

To approximate the self-energy contribution due to disorder scattering we use the full T-matrix calculated at first order in impurity density n_{imp} (i.e. the non-crossing approximation). Within

this approximation the self-energy, which is also a matrix, is [7]

$$[\boldsymbol{\Sigma}]_{\alpha\beta}(\omega) = n_{\mathrm{imp}} u_0 \left\langle \left(\mathbb{1} - \frac{u_0}{2\pi R_{\mathrm{w}}} \sum_{\ell=\pm\frac{1}{2},\pm\frac{3}{2},\ldots} \int \frac{dk}{2\pi\hbar v_F} \boldsymbol{G}^0(k,\mu,\ell) \right)^{-1} \right\rangle_{\mathrm{imp}} \tag{10}$$

$$= n_{\mathrm{imp}} u_0 \left(\mathbb{1} - u_0 \sum_{\ell=\pm\frac{1}{2},\pm\frac{3}{2},\ldots} \Delta(\mu,\ell) \right)^{-1} .$$

For small δ the $\boldsymbol{\Delta}(\mu,\ell)$, from Eq. (6), only has a substantial imaginary part for $|\mu| > |\varepsilon_\ell|$, this means that a conductivity channel only contributes when the corresponding sub-band has an occupation of electrons (holes) for the upper (lower) Dirac cone. Further the off-diagonal components of $\boldsymbol{\Sigma}$ are exactly zero for diagonal impurities since the off-diagonal contributions of positive and negative ℓ cancel.

The self-energy can also be calculated self-consistently by replacing $\mu \to \mu + \Sigma_{11}$ and $\varepsilon_\ell \to \varepsilon_\ell + \Sigma_{12}$, where Σ_{11} and Σ_{12} are the diagonal and off-diagonal components, respectively. Self-consistency has a similar impact on the self-energy as δ, that is, the breadth of the peaks in scattering rate become broadened in proportion to the self-energy itself. Ultimately this means that peaks at high sub-band index become "washed out" when the self-energy is of the same order of magnitude as the sub-band spacing. This is not of relevance for the experimental situation discussed here where the mean free path is several times the wire radius, but would limit the number of peaks seen in thicker or dirtier wires where the ratio of mean free path and radius can be substantially smaller.

Supplementary Note 10. Conductivity calculation

The conductivity is found using the Kubo formula to calculate the current-current correlation function in linear response. The current operator along the wire is given by $j_x = e\partial H/\partial k = -i\hbar v_F e \sigma_y$. We neglect vertex corrections which previous studies have shown only have a quantitative effect on overall conductivity[7]. Within this scheme the DC conductivity is given by

$$\sigma = \frac{e^2 v_F^2 \hbar^2}{\pi} \frac{1}{2\pi R_{\mathrm{w}}} \sum_{\ell=\pm\frac{1}{2},\pm\frac{3}{2},\ldots} \int d\omega\, n_F'(\omega) \int \frac{dk}{2\pi\hbar} \mathrm{Tr}\left(\sigma_y \boldsymbol{G}(\omega,k,\ell) \sigma_y \boldsymbol{G}^\dagger(\omega,k,\ell) \right) = \sum_{\ell=\pm\frac{1}{2},\pm\frac{3}{2},\ldots} \sigma_\ell(\mu), \tag{11}$$

where in general the contribution σ_ℓ is given by

$$\sigma_\ell(\mu) = \frac{e^2 v_F}{(2\pi)^2 R_{\mathrm{w}}} \frac{1}{\tilde{\mu}\Gamma_{11} - \tilde{\varepsilon}_\ell \Gamma_{12}} \mathrm{Im} \left\{ \frac{\lambda}{\sqrt{\xi}} - \frac{\lambda^\dagger}{\sqrt{\xi^\dagger}} \right\} \approx \theta(\mu^2 - \varepsilon_l^2) \frac{e^2 v_F}{2\pi^2 R_{\mathrm{w}} |\Gamma_{11}|} \frac{\mu^2 - \varepsilon_l^2}{\mu\sqrt{\mu^2 - \varepsilon_l^2}}, \tag{12}$$

where $\mathbf{\Gamma} = \mathrm{Im}\mathbf{\Sigma}$ is the (spin-resolved) scattering rate, $\tilde{\mu} = \mu + \mathrm{Re}\Sigma_{11}$, $\tilde{\varepsilon}_\ell = \varepsilon_\ell + \mathrm{Re}\Sigma_{12}$, and the factors $\xi = -(\mu + i\Gamma_{11})^2 + (\varepsilon_\ell + i\Gamma_{12})^2$ and $\lambda = -\tilde{\varepsilon}_\ell^2 - i\tilde{\varepsilon}_\ell\Gamma_{12} + i\tilde{\mu}\Gamma_{11} + \tilde{\mu}^2$ ensure the channel only contributes to conductivity when $|\mu| \gtrsim |\varepsilon_\ell|$. The approximation in Eq. (12) is valid for small self-energies and diagonal impurities.

Supplementary Note 11. Parameters for theory plots

An important parameter for our theory is u_0, the strength of the impurity potential. In Supplementary Fig. 5 we show the resistivity as function of gate voltage for weak, intermediate and strong impurity potentials. Only for weak impurity potentials, $\frac{u_0}{\hbar v_F R_\mathrm{w}} \ll 1$, peaks occur at the band edges and particle- and hole doping are almost equivalent. For intermediate impurity strength, $\frac{u_0}{\hbar v_F R_\mathrm{w}} \sim 1$, the curve is highly asymmetric. For $\frac{u_0}{\hbar v_F R_\mathrm{w}} \gg 1$, the particle-hole symmetry is restored but instead of peaks one obtains dips.

We find that only weak u_0 fit the experimental data. This is expected because the wavefunction of the conduction channel wraps around the circumference of the wire, effectivly diluting the impurity by a factor $1/(2\pi R_\mathrm{w})$. To be precise: Weak scattering can be defined by $u_0\bar{\rho} \ll 1$, where $\bar{\rho} \sim \frac{1}{2\pi v_F \hbar R_\mathrm{w}}$ is the typical density of states in the system for small ℓ. Assuming that the width of the scattering potential is of the order of the lattice constant a, the weak scattering limit is reached when the amplitude, $V_0 = u_0/a^2$, of the scattering potential fulfils $V_0 \ll \frac{v_F}{a\hbar}\frac{2\pi R_\mathrm{w}}{a}$. Since in our experiments the circumference is many lattice constants, $\frac{R_\mathrm{w}}{a} \sim 100$, the weak scattering limit is generically realised.

Therefore, for the plots in Fig. 2 and Fig. 3 of the main text we use $u_0 = 0.1 v_F \hbar R_\mathrm{w}$, $n_\mathrm{imp} = 20/R_\mathrm{w}^2$ and $\delta = 0.025 v_F \hbar/R_\mathrm{w}$, see also method section. This reproduces the order of magnitude ~ 5 nm/kΩ of the fluctuations in wire conductivity found experimentally.

While the main contribution to the peaks at the sub-band edges arised from the diverging density

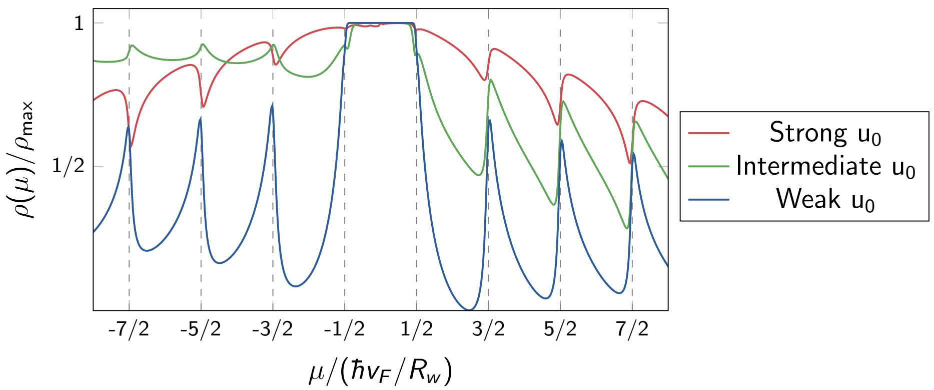

Supplementary Fig. 5: **Resistivity as a function of chemical potential in the weak, intermediate, and strong scattering regimes**. A weak scatterer, $\frac{u_0}{\hbar v_\mathrm{F} R_\mathrm{w}} = 0.1$, $n_{imp} = 20/R_\mathrm{w}^2$, as in the main text, is shown as the blue curve. The green curve, $\frac{u_0}{\hbar v_\mathrm{F} R_\mathrm{w}} = 1$, $n_{imp} = 2/R_\mathrm{w}^2$, describes scatterers of intermediate strength, the red curve, a strong scatterer $\frac{u_0}{\hbar v_\mathrm{F} R_\mathrm{w}} = 20$, $n_{imp} = 0.1/R_\mathrm{w}^2$. Strong scattering is capable of turning resistivity peaks at sub-band edges into resistivity dips, however such a situation is unlikely (see text).

of states, this effect is further enhance by a matrix-element effect closely related to the topological protection of TI surface states. Here the spin orientation is locked to the propagation direction and scattering from $\boldsymbol{k}$ to $-\boldsymbol{k}$ is prohibited by time-reversal symmetry. In our nanowires, $k_y = \ell/R_\mathrm{w}$ is quantized, and the 1D bands are doubly degenerate as for each $k_x = k$, two transverse momenta, $\pm k_y$, are possible. For $k_x \gg k_y$, however, the 2D vectors $\boldsymbol{k} = (k_x, \pm k_y)$ and $\boldsymbol{k} = (-k_x, \pm k_y)$ are almost antiparallel, leading to a suppression of the scattering rate by the factor $(k_y/k_x)^2$. This matrix element effect (encoded in the 2×2 matrix structure of the T-matrix) substantially suppresses backscattering among occupied channels with $\ell/R_\mathrm{w} \ll k_x$ relative to the scattering to a newly opened channel with a large k_y.

Supplementary Note 12. Electron-hole puddles

Several physical effects have not been taken into account by our theoretical treatment. For example, we do not reproduce the type II fluctuations which likely arise from extra charge traps in the experimental setup. We also assume scattering from local impurities, but there will be also long-range potentials arising from randomly distributed positive and negative charges in our compensation doped samples of $(Bi_{1-x}Sb_x)_2Te_3$. In bulk systems, these lead to the formation of electron-hole puddles [8, 9] both in the bulk and in the surface states [10] of TIs. Estimates from Ref. 11 suggest that bulk puddles are absent in TI nanowires with a radius of less than $\sim 100\,\mathrm{nm}$. But also in this case, one can expect substantial fluctuations of the chemical potential on the surface of the TI. For surface states of bulk samples of $BiSbTeSe_2$, fluctuations of the local chemical potential with an amplitude of 20 meV on a length scale of 50 nm have been measured by scanning tunneling spectroscopy [10]. While the size of these fluctuations will be reduced for nanowires, surface puddles can be expected to be of quantitative importance (perhaps, also for type II fluctuations).

Supplementary Note 13. Electrostatic modeling and anisotropy effects

In Supplementary Notes 7-11 above, we investigated disorder effects assuming an idealized setup with cylindrical symmetry. Now we investigate the role of anisotropy effects arising both from the hexagonal shape of the wire and of the electrostatic environment. As shown in Supplementary Fig. 6a, wires with diameters between 29 and 43 nm are positioned on a 255 nm thick dielectric SiO_2 substrate, see methods for details. When in our experiment the chemical potential is adjusted via a flat bottom gate below the dielectric substrate, the induced charge around the wire will not be uniform and will depend on both the substrate/gate geometry and also the approximately hexagonal wire shape. These geometric effects can introduce changes to both the sub-bands and the charging physics of the wires. We will show that Klein-tunnelling physics means that the locations of the sub-band minima in terms of chemical potential are largely unchanged and so the underlying theoretical signature of confinement: An increased scattering at the bottom of each sub-band and a corresponding resistivity peak, should remain robust for most set-ups. In contrast, charging effects could be important such that the mapping from induced charge density to chemical

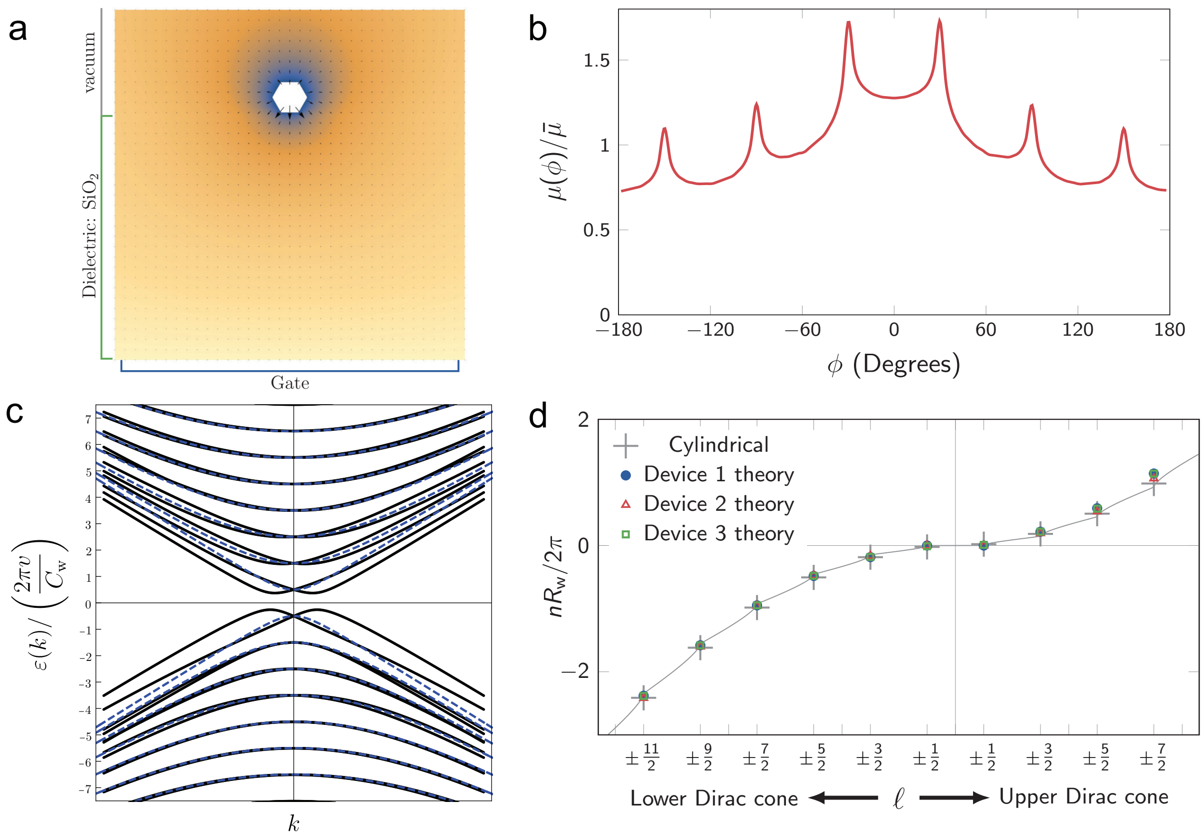

Supplementary Fig. 6: **Effect of a bottom gate on an hexagonal nanowire**. **a** Numerical solution of the Laplace equation and electric field due to bottom gate and metallic hexagonal wire. **b** Ratio of chemical potential, $\mu(\phi)$, to the average chemical $\bar{\mu}$ around the nanowire. **c** Resulting sub-bands of device 1 when tuned to the charge neutrality point. The sub-bands of an isotropic circular wire are shown as blue dashed lines. **d** Theoretically calculated locations of the van-Hove singularities in the experiment as a function of charge density for anisotropic hexagonal wires (circles) compared to the isotropic wire (grey crosses).

potential is influenced by the geometry of the system.

For our experiment, however, we will argue that these effects are below the resolution of the experiment and so the cylindrical wire description above remains a valid approximate model of our experimental set-up. Such effects could, however, be important in future experiments.

We would like to stress that, as discussed above, the following electrostatic modeling is not fully quantitative as it ignores, for example, parasitic capacitances as arising, e.g., from the gold contacts, see Fig. 1d of the main text, and the finite length of the wire. Furthermore, our modeling assumes metallic surfaces of the topological insulator and there are effects arising from the finite penetration depth of the surface state, the experimental uncertainty in the radius of the wire and its surface chemistry, and also possible effects arising from electron-hole puddles, see Supplementary Note 12, which may act as extra charge reservoirs.

Supplementary Note 14. Influence of geometry on energies of sub-band minima

We start by addressing the influence of the geometric effects on the sub-band minima and associated resistivity maxima. An important question for our experiment and physical interpretation is the consequence the induced charge anisotropy has for the confinement of surface states around the wire. It turns out that the Dirac nature of the TI surface states leads to Klein tunnelling of the state through any smooth surface potential and that, as a result, the energies of sub-band minima and associated peaks in resistivity are only very weakly sensitive to any charge anisotropy.

The charge anisotropy induced by bottom gating can be modelled as an angular dependent chemical potential $\mu(\phi) = \bar{\mu} + \delta\mu(\phi)$, where $\bar{\mu}$ is the averaged chemical potential around the wire and $\delta\mu(\phi)$ the angular variation of the chemical potential. (An estimate of $\mu(\phi)$ calculated using an electrostatic model and the full influence on sub-bands, which is important for the charge density, can be found below).

Of relevance to our study is the impact this angular dependent potential has on the quantisation of the surface states. In the cylindrical case the location of the quantisation peaks is set by the energies of the bottom of the sub-bands i.e. at $k = 0$. Adding an inhomogeneous chemical potential $\mu(\phi)$ to the Hamiltonian of the surface at the sub-band bottom gives

$$H_{k=0} = i\hbar v_F \sigma_x \frac{\partial_\phi}{R_W} + \mu(\phi)\mathbb{1}. \tag{13}$$

This Hamiltonian still has quantum confined eigenstates around the wire, given exactly by

$$\psi_\pm(\ell, k=0) = \boldsymbol{\xi}_\pm e^{i\left((\ell-\bar{\mu})\phi - \int_0^\phi d\phi'\, \delta\mu(\phi')\right)}, \tag{14}$$

with $\ell = \pm\frac{1}{2}, \pm\frac{3}{2}, \pm\frac{5}{2}, ...$ and the spinors $\boldsymbol{\xi}_\pm$ are eigenspinors of the Pauli-matrix σ_x. Although the states $\psi_\pm(\ell, k=0)$ are no longer simultaneous eigenstates of the angular momentum operator $L_z = -iv_F\frac{\partial_\phi}{R_W}$, they still have eigen-energies $\varepsilon_{\ell,\pm} = \pm\hbar v_F|\ell|$, the same as those of a wire with an isotropic surface chemical potential $\bar{\mu}$. Importantly this means that the locations where the chemical potential are tuned to a sub-band edge, $\mu_0 = \varepsilon_{\ell,\pm}$, will be very close to those of an idealised circular wire without any inhomogeneity.

Physically, the absence of an effect at $k = 0$ is due to Klein tunnelling of 1d chiral modes around a wire through the additional potential $\delta\mu(\phi)$, this potential only induces an angular dependence of the mode's phase and does not alter its energy at $k = 0$ [12]. Away from $k = 0$ the Klein-tunneling is not perfect and anistotropies can cause small shifts in the sub-band minima to finite k, most prominently for low angular momenta sub-bands, see Supplementary Fig. 6c and explanation below. Strikingly, however, the perfect Klein-tunnelling at $k = 0$ and strong but imperfect Klein-tunnelling for small k means that there is very little change in the energies where sub-band minima occur and associated resistivity peaks at these energies, even if that anisotropy is much larger than the sub-band gap.

Supplementary Note 15. Estimate of the anisotropic chemical potential

Although Klein-tunnelling results in extremely small changes in the energies of sub-band minima due to geometric effects, the charge density – as changed in the experiment – will be dependent on these effects due to changes at large k where Klein-tunnelling is less effective and suppressed [12]. To assess the influence of these effects on density we first need to obtain an estimate of the induced charge and resulting anisotropy in chemical potential $\mu(\phi) = \bar{\mu} + \delta\mu(\phi)$, this can be approximated using a classical electrostatic analysis. As in Ref. [12], the inhomogeneity of charge density can be estimated by numerically solving the Laplace equation for our set-up, $\nabla\left[\varepsilon(x,y)\nabla\Phi(x,y)\right] = 0$, as shown in Supplementary Fig. 6. We use $\varepsilon = 3.5$ for the dielectric constant of SiO_2 and the Dirichlet conditions $\Phi(x,y) = V_G$ on the gate and $\Phi(x,y) = 0$ on the surface of the TI nanowire, which assumes the wire is perfectly metallic and hexagonal (with rounded corners, see below). The induced charge on the nanowire is proportional to the electric field normal to the wire, $\sim \nabla\Phi(x,y)\cdot\hat{\boldsymbol{r}}$. To obtain an estimate for $\mu(\phi)$, we can use the 2D Dirac relation $n \propto \mu^2$. The ratio of the chemical potential to its average is shown Supplementary Fig. 6b in terms of the polar angle around the wire. We see that the flat bottom gate leads to an imbalance in chemical potential around the wire and that the hexagonal shape results in sharp features at the corners of the wire. This numerical estimate assumes that the surface of the nanowire is perfectly metallic, with no penetration of the surface state into the wire. The influence of the finite penetration of the surface state into the wire will be most apparent at the corners, which cannot anyway be treated as perfectly sharp, and at the bottom of the wire, where the wire touches the dielectric. To model this we assume a small distance of 1 nm between dielectric and bottom of the wire and round the edges of the hexagon by the equivalent of 0.2 nm, which is much smaller than estimates for the penetration depth of the surface state and so likely overestimates the sharpness of the voltage spikes at the corners.

Supplementary Note 16. Influence of geometry on charge density of sub-band minima

Having obtained an upper bound for the chemical potential anisotropy around the wire, $\delta\mu(\phi)$, we can use this to calculate the full change of the resulting sub-bands through the matrix elements $\langle \ell,\eta|\,\delta\mu(\phi)\,|\ell',\eta'\rangle$ between different sub-bands, where ℓ is the angular momentum and $\eta = \pm$ indicates particle/hole sub-bands. These matrix elements can be simplified since

$$\langle \ell,\eta|\,\delta\mu(\phi)\,|\ell',\eta'\rangle = \boldsymbol{\xi}^{\dagger}_{\ell,\eta}(k)\boldsymbol{\xi}^{\dagger}_{\ell',\eta'}(k)\int \frac{d\phi}{2\pi}e^{i(\ell'-\ell)\phi}\delta\mu(\phi) = \left[\boldsymbol{\xi}^{\dagger}_{\ell,\eta}(k)\boldsymbol{\xi}_{\ell',\eta'}(k)\right]\delta_{|\ell-\ell'|,n}\frac{\delta\mu_{(n)}}{2} \qquad (15)$$

where the product in square brackets accounts for Klein-tunnelling effects and we have used the Fourier cosine transformation of the anisotropic chemical potential $\delta\mu(\phi) = \sum_{n=1}^{\infty}\delta\mu_{(n)}\cos(n\phi)$. Diagonalising the full Hamiltonian matrix for each k we can obtain the band structure for a given average chemical potential $\bar{\mu}$. An example of the resulting sub-bands is shown in Supplementary Fig. 6c which is calculated for the parameters of device 1 when it is tuned to the Dirac point. As expected from the above discussion on Klein tunnelling, there is little alteration to the locations

of the sub-band minima in terms of energy apart from a small splitting of the lowest angular momenta sub-band $\ell = \pm 1/2$ away from $k = 0$, which results from the fact $\delta\mu_{(1)}$ is largest. Since this sub-band is anyway not visible in our experiment the theoretical discussion of transport in the main text is essentially unchanged by geometric effects. In particular, the theoretical plot Fig. 3 of the main text, which shows that resistivity peaks are equally spaced as a function of chemical potential for a circular wire, remains valid for our geometry apart from very small shifts in peak positions and the onset of a finite conductivity at a slightly smaller chemical potential.

Despite this, we see in Supplementary Fig. 6c that for large k the bands and their net velocity are affected by the anisotropies arising from $\mu(\phi)$. Thus the charge density $n(\bar{\mu})$ is modified, which is the quantity controlled in our experiment by the gate voltage. To estimate the influence of the anisotropy on the position of the peaks as a function of charge density – rather than chemical potential – we show in panel Supplementary Fig. 6d the charge density at which the bottom of a sub-band occurs and a resistivity peak is expected (to be compared to Fig. 2d of our paper). The plot is calculated self-consistently by tuning the chemical potential to a given sub-band minimum, then recalculating all bands for the applied potential. This allows the recalculation of densities and applied voltages in an iterative procedure until convergence is reached.

Supplementary Fig. 6d shows the resulting effect is small for all three of our experimental devices, which differ by the amount of gate voltage (V_G^0 = 30.5, 10, and 18.5 V for device 1, 2, and 3, respectively) needed to reach charge neutrality. For negative voltages the effects are completely negligible for all three devices but for positive voltages which add to V_G^0 there is a small effect visible, most pronounced for device 1 as V_G^0 is largest in this case. More precisely, we find that the change of density Δn is smaller than $0.2\frac{2\pi}{R_W}$ which corresponds to a change of gate voltage of $\approx 4.5\,\mathrm{V}$ for the largest peak shift of device 1, i.e. $\ell = 7/2$ for $\Delta V_G > 0$. Actually, this trend is consistent with our data (the circular-wire fits predict the position of the peak at $\Delta V_G \approx 24\,\mathrm{V}$ but it occurs experimentally at $\Delta V_G \approx 27\,\mathrm{V}$). Although this might indicate the change of charge density is just visible in our experiment, one should also take into account experimental noise, the substantial uncertainties in our electrostatic modeling, and further small perturbations to the sub-band structure, e.g. due to non-linearities of the Dirac spectrum.

For device 2 the effect of anisotropies is smaller compared to device 1, while device 3 is similar to device 1. In device 2 we find a maximal shift in density of $0.12\frac{2\pi}{R_W}$ or $\approx 2.5\,\mathrm{V}$ for the largest shift in a peak, i.e. for $\ell = 7/2$ of the upper Dirac cone, which occurs at $\Delta V_G = 28\,\mathrm{V}$. We therefore conclude that the effects of gate-, dielectric- and shape-induced anisotropies on charge density are small for our experimental set-up and do not affect our modelling of the experiment. In particular the presence of quantum-confined surface states in a bulk-insulating quantum wire identified by resistivity peaks with a super-linear dependence on gate voltage remains valid our set-up, but geometric effects may be relevant for future devices, in particular when a large gate voltage is necessary for accessing the Dirac point.

---

[1] Virk, N. & Yazyev, O. V. Dirac fermions at high-index surfaces of bismuth chalcogenide topological insulator nanostructures. *Sci. Rep.* **6**, 20220 (2016).

[2] Zhu, H. *et al.* Topological insulator Bi_2Se_3 nanowire high performance field-effect transistors. *Sci. Rep.* **3**, 1757 (2013).

[3] Kong, D. *et al.* Topological insulator nanowires and nanoribbons. *Nano Lett.* **10**, 329–333 (2010). 0912.5045.

[4] Zhang, J. *et al.* Band structure engineering in $(Bi_{1-x}Sb_x)_2Te_3$ ternary topological insulators. *Nat. Commun.* **2**, 574 (2011).

[5] Wunnicke, O. Gate capacitance of back-gated nanowire field-effect transistors. *Appl. Phys. Lett.* **89**, 083102 (2006).

[6] Zhang, Y. & Vishwanath, A. Anomalous Aharonov-Bohm conductance oscillations from topological insulator surface states. *Phys. Rev. Lett.* **105**, 206601 (2010).

[7] Taskin, A. *et al.* Planar hall effect from the surface of topological insulators. *Nat. Commun.* **8**, 1340 (2017).

[8] Borgwardt, N. *et al.* Self-organized charge puddles in a three-dimensional topological material. *Phys. Rev. B* **93**, 245149 (2016).

[9] Breunig, O. *et al.* Gigantic negative magnetoresistance in the bulk of a disordered topological insulator. *Nat. Commun.* **8**, 15545 (2017).

[10] Knispel, T. *et al.* Charge puddles in the bulk and on the surface of the topological insulator $BiSbTeSe_2$ studied by scanning tunneling microscopy and optical spectroscopy. *Phys. Rev. B* **96**, 195135 (2017).

[11] Bömerich, T., Lux, J., Feng, Q. T. & Rosch, A. Length scale of puddle formation in compensation-doped semiconductors and topological insulators. *Phys. Rev. B* **96**, 075204 (2017).

[12] Ziegler, J. *et al.* Probing spin helical surface states in topological HgTe nanowires. *Phys. Rev. B* **97**, 035157 (2018).
